# Integrative Analysis of MAPK14 as a Potential Biomarker for Cardioembolic Stroke

**DOI:** 10.1155/2020/9502820

**Published:** 2020-08-17

**Authors:** Zhao Li, Li Xu, Qingxiu Wang

**Affiliations:** ^1^Department of Anesthesiology, East Hospital, Tongji University School of Medicine, Shanghai 200120, China; ^2^Department of Anesthesiology, The First People's Hospital of Changde, Changde 415000, China

## Abstract

The aim of this study was to obtain the candidate genes and biomarkers that are significantly related to cardioembolic stroke (CS) by applying bioinformatics analysis. In accordance with the results of the weighted gene coexpression network analysis (WGCNA) in the GSE58294 dataset, 11 CS-related coexpression network modules were identified in this study. Correlation analysis showed that the black and pink modules are significantly associated with CS. A total of 18 core genes in the black module and one core gene in the pink module were determined. We then identified differentially expressed genes (DEGs) of CS at 3 h, 5 h, and 24 h postonset. After performing intersection, it was found that 311 genes were coexpressed at these three time points. These genes were majorly enriched in positive regulation of transferase activity and regulation of peptidase activity. The abovementioned coexpressed DEGs were subjected to protein-protein interaction analysis and subnetwork module analysis. Subsequently, we used cytoHubba to obtain 11 key genes from DEGs. The intersection of the core genes screened from WGCNA and the key genes selected from DEGs yielded the MAPK14 gene. The expression level of MAPK14 on the receiver operating characteristic (ROC) curves of CS at 3 h, 5 h, and 24 h showed that the area under the ROC curve (AUC) was 0.923, 0.934, and 0.941, respectively. In a nutshell, MAPK14 screened out by using WGCNA showed differential expression in CS. We conclude that MAPK14 can be used as a potential biological marker of CS and exhibits potential to predict the physiopathological condition of CS patients.

## 1. Introduction

Stroke is still a devastating neurological disease, the second leading cause of death and the leading cause of severe disability and physical impairment around the world [1]. The global burden of stroke has been exacerbating year by year [2]. According to the TOAST criteria, stroke can be mainly divided into large artery atherosclerosis (LAA), small-artery occlusion (SAO), cardioembolism (CE), stroke of other demonstrated etiology (SOE), and stroke of other undemonstrated etiology (SUE) [3]. The accurate etiological classification of stroke can help determine the optimal, systematic, and individualized diagnosis and therapeutic plan, thereby achieving early diagnosis, early treatment, early rehabilitation, and early prevention of relapse. Therefore, it is of critical relevance to improve the treatment effect and prognosis of patients with stroke.

Cardioembolic stroke (CS) patients account for around 20% of all patients with acute ischemic stroke [4]. Atrial fibrillation (AF) is the most common risk factor that contributes to CS and makes up to about 50% of CS events [5]. Moreover, as the global population continue to age, the proportion of patients with CS is likely to increase further, imposing an increasingly heavy burden on the individual, medical care, and socioeconomic levels [6]. A fraction of CS patients can improve their prognosis and prevent the recurrence of stroke by undergoing anticoagulation therapy [7]. However, less than one-third of these CS patients with indications for anticoagulation have received the correct treatment [8].

The diagnosis of some CS patients proves difficult, requiring further selection and improvement of a series of auxiliary examinations to figure out the evidence for etiology [9]. Some inspection items are expensive and require long appointment lead time and therefore are not broadly allocated in hospitals at all levels [10]. The patients cannot complete the tests within a short time after admission, so it is hard to perform early diagnosis and classification of stroke based on TOAST etiology, consequently leading to augmented proportion of unknown causes and compromised the efficacy of early guidance on the clinical treatment [10]. Therefore, CS patients pose a daunting challenge to the treatment of acute ischemic stroke. Finding the accurate biomarkers and clarifying the potential biological mechanisms are of critical importance for the early diagnosis and effective therapeutic intervention of CS. Studies have identified the correlation between natriuretic peptides and cardiac stroke and that LP-PLA2 level has been found to be associated with greater severity and risk in large artery atherosclerosis stroke [11, 12]. However, the accurate biomarkers approved for diagnosis and treatment of CS have not been well defined.

Thanks to the swift advance of biotechnology in recent years, especially the development of next-generation sequencing technology, the amount of biological data has been growing at an explosive rate, making it less practicable to analyze these data by employing traditional data analysis methods [13]. The emergence and development of high-throughput sequencing technology has revolutionized biological research, ushering in the biological network analysis methods developed on the basis of the complex network theory of high-throughput data [14]. These methods can be used to systematically describe and analyze these high-throughput data. Among them, the gene coexpression network plays a critical role in biological research, and one of the representative method is the weighted gene coexpression network analysis (WGCNA) [15].

WGCNA has been broadly used in various disciplines of biological research, such as development and disease [16, 17]. However, the use of WGCNA to construct a scale-free network based on the gene expression pattern of CS remains scarce. In the current study, WGCNA was used to analyze the CS expression profile data in the public database, in an attempt to identify the CS-related gene modules. Furthermore, the core genes in the module were excavated to ascertain the biomarkers highly correlated with the diagnosis of CS. Moreover, functional enrichment analysis and other bioinformatics methods were combined to provide new ideas and methods for the diagnosis and intervention of CS.

## 2. Material and Methods

### 2.1. Microarray

To our certain knowledge, GEO is the largest public gene expression database developed by NCBI [18]. The gene annotation file and the gene matrix text file in the CS-related gene chip GSE58294 were obtained from the NCBI GEO database. The samples in this series can be subdivided into four categories, including 23 healthy samples, 23 CS samples of 3 hours postonset, 23 CS samples of 5 hours postonset, and 23 CS samples of 24 hours postonset. The genomic annotation platform for GSE58294 is GPL570 (Affymetrix Human Genome U133 Plus 2.0 Array; Affymetrix, Santa Clara, CA, USA). The probes in the obtained gene expression profile were converted into gene symbols through the annotation files on the platform GPL570. The probes that correspond to multiple genes were removed from the study; for the gene with multiple probes, the expression value of such gene was averaged based on these probes.

### 2.2. Construction of Coexpression Network

WGCNA package in R was used to perform the coexpression network analysis. For starters, we conducted a cluster analysis on the expression data in the samples and deleted the outliers. Subsequently, the pickSoftThreshold function was applied to select an appropriate soft threshold power *β*, which is a criterion based on the scale-free topology. The scale-free topological exponential curve fitting became flatter after reaching peak, indicating that the selected soft threshold needs greater average connectivity.

The pairwise correlation matrix of each group of genes was calculated, and then, the soft threshold was referred to convert the similarity matrix into an adjacency matrix. Afterwards, the hierarchical clustering tree was drawn to unmask the hierarchical clustering. Given that the branches of the hierarchical clustering tree are densely interconnected, the dynamic tree cut method was used to cut the branches and to divide the genes into different modules. The thresholds for the sizes of minimum module and merged module were set to 30 and 0.25, respectively. Those which were poorly connected with other genes would be assigned into the grey module and not used for the subsequent analysis.

### 2.3. Correlation Analysis and Identification of CS-Related Modules

The genes were divided into different modules based on the results of the WGCNA analysis [15], and subsequently, we calculated the correlation coefficients between different modules. A heat map was plotted to visualize the correlation. In order to detect the significant modules associated with CS, the correlation between clinical factors of CS and the modules was evaluated using the WGCNA package in R. Module eigengenes (MEs) were the major component of each module and represent the overall expression level of the corresponding gene. Modules with high correlation were screened out by assessing the correlation coefficient between MEs and clinical traits (disease status and onset time) (*p* < 0.05). Gene significance (GS) was defined to determine the association of gene expression with external traits, while module membership (MM) was adopted to evaluate the correlation between each intramodular gene and ME. The modules proved most susceptible to CS were further verified by analyzing the correlation between GS and MM. Then, GS and MM were used to identify the hub genes in CS-related modules.

### 2.4. Differentially Expressed Gene (DEG) Screening

In light of the microarray data of healthy individuals and CS patients in GSE58294, we screened these genes for DEGs within 3 h, 5 h, and 24 h after onset of CS by using GEO2R. The screening conditions were adj. *p*.val < 0.05 and ∣log_2_ FC | >1. A Venn diagram was plotted to screen out the common DEGs after different hours of CS onset.

### 2.5. The Identification of Key Genes in DEGs

The functional enrichment analysis of these common DEGs was conducted by using the Metascape database [19]. Subsequently, these obtained common DEGs were employed to construct the protein-protein interaction (PPI) network by using the STRING database, followed by visual analysis by using Cytoscape 3.7.2 [20]. The MCODE plug-in (parameters: degree cutoff: 2, node score cutoff: 0.2, *K*-core: 2, and max. depth: 100) was used to display the modules in the PPI network. Afterwards, we used the five algorithms in the Cytoscape plugin cytoHubba to screen out the hub genes [21].

### 2.6. Statistics

Statistical analysis of gene expression data was conducted by using GraphPad Prism 8.3.0 or R 3.7.2. Meanwhile, both two software were used for image plotting. The *t*-test was used to analyze the difference in the mean value between the two groups. The receiver operating characteristic curve (ROC) was schemed to evaluate the diagnostic accuracy of genes. The sensitivity and specificity of genes were investigated by using AUC. For all analyses, *p* < 0.05 was considered to indicate statistical significance.

## 3. Results

### 3.1. Construction of Coexpression Network

The expression data files of the GSE58294 dataset and the annotation file of GLP570 were downloaded from the NCBI GEO database. To establish the coexpression network, the expression data of CS patients and healthy controls were extracted from GSE58294, and the probes in the original expression data file were converted into 21,654 genes. Afterwards, the SD value of each gene was quantified, and then, the genes were ranked in a descending order. The top 25% on the list, including 5,414 genes, were enrolled for subsequent WGCNA analysis.

**Figure 1:**
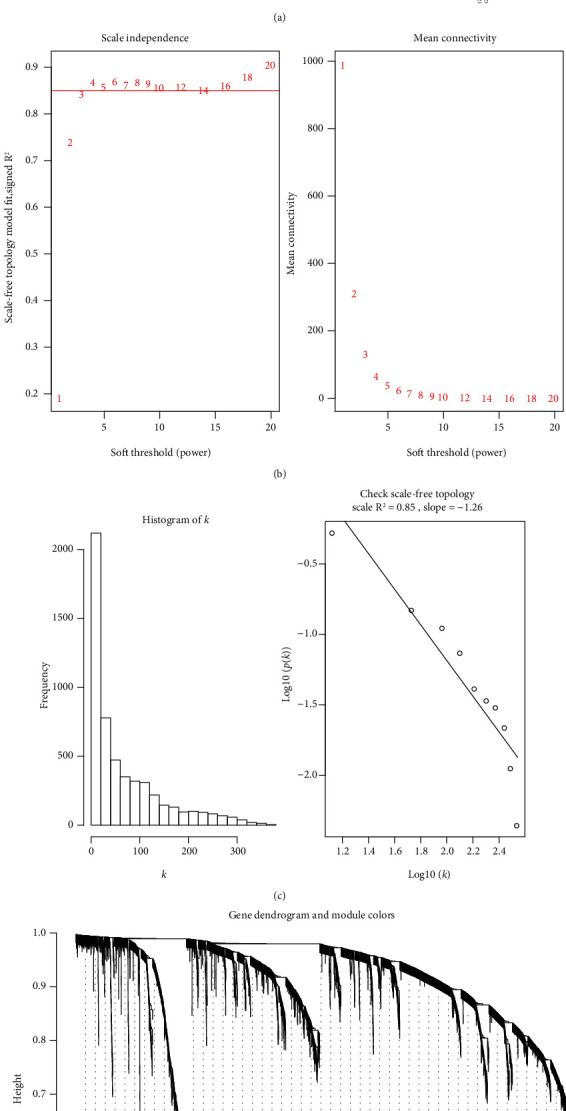
Construction of coexpression network in GSE58294. (a) Clustering dendrogram of samples from GSE58294. (b) Analysis of the scale-free fit index and the mean connectivity based on various soft thresholding powers (*β*). (c) Histogram of connection distribution and scale-free topology when *β* = 4. (d) Clustering dendrograms of genes based on TOM-based dissimilarity.

To detect the possible outlier samples, a cluster tree consisting of 92 samples was constructed by using flashClust package in R (Figure 1(a)). As a result, no outlier was found in the samples included in the WGCNA analysis. To better build a scale-free network distribution, the pickSoftThreshold function was applied to determine the appropriate soft threshold (*β*) in WGCNA. 1-20 thresholds were selected for each sample included in the WGCNA analysis, so as to calculate the scale-free topology index separately (Figure 1(b)). In the event of *β* = 4, the square of the correlation coefficient of log(*k*) and log(*p*(*k*)) became greater than 0.85 (Figure 1(c)). Meanwhile, the average network connectivity corresponding to this threshold was close to zero, indicating that the network connectivity at that time was rather low, approaching to the condition of a scale-free network. Therefore, *β* = 4 was selected as the soft threshold to construct the gene coexpression network.

### 3.2. Visualization of Gene Modules

The value of soft threshold *β* was set to 4, and then, we constructed a hierarchical clustering tree to identify the gene coexpression network following the procedures of WGCNA. In the current study, we used the dynamic tree cut to identify the gene modules, and the minimum number of genes in the module was set to 30. The modules were ranked based on the number of variables contained in the modules. 15 corresponding modules were initially obtained, as shown in Figure 1(d). Different colors represent different modules. Among them, the genes in the grey modules are the ones that have not been housed to any other modules.

**Figure 2:**
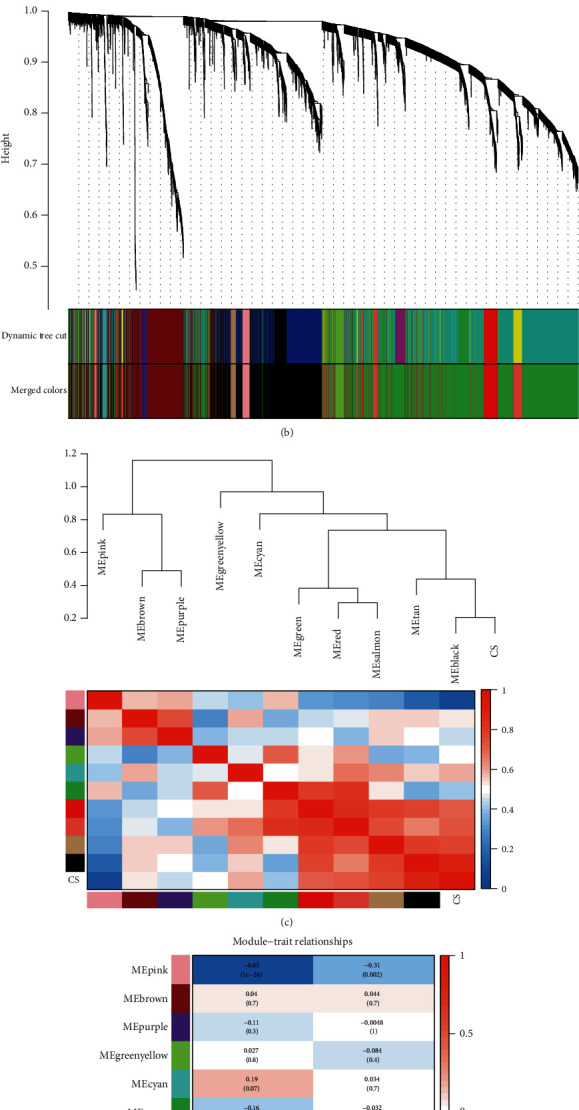
The correlation between modules and CS. (a) Clustering dendrogram of modules based on dissimilarity measure. (b) Clustering dendrograms of genes after modules were merged. (c) Clustering dendrogram and adjacency heatmap of module eigengenes. (d) Heatmap of the module-trait relationships. CS: cardioembolic stroke. Time presents postonset time following CS.

To assess the coexpression similarity of the module, the characteristic genes of each module were calculated and clustered in accordance with their respective correlation coefficients. The threshold of mergeCutHeight was set to 0.25 (Figure 2(a)). After merging the modules, the diagram of gene tree was plotted again. The original colors of the modules and the colors of the merged modules were indicated in the diagram (Figure 2(b)).

### 3.3. Identification of CS-Related Key Modules

To further investigate the correlation between various gene modules, we calculated the correlation coefficients between the modules. Subsequently, the clustering tree and the heat map were schemed to visualize the association between the modules (Figure 2(c)). These modules clustered into the same general category showed a similar expression with respect to the genes.

In order to ascertain the genetic modules that are related to CS, we extracted the clinical information of the sample from GSE58294. Subsequently, the correlations between the specific modules, which were labeled by with different colors, and two clinical characteristics (disease status and onset time) were investigated. The analysis results showed that the black module and pink module are the two gene modules significantly related to CS (Figure 2(d)). The correlation coefficient of the black module and CS was 0.8, indicating that the genes in this module are likely to play an important role in the progression of CS. Moreover, the correlation coefficient between the pink module and CS being -0.85 indicated that such module was negatively correlated with the status of CS. Thus, it was rational to select the black module and pink module as the key modules of CS. Through more detailed analysis into the genes in the modules, CS-related core genes could be verified.

### 3.4. Screening of CS-Related Core Genes Using WGCNA

**Figure 3:**
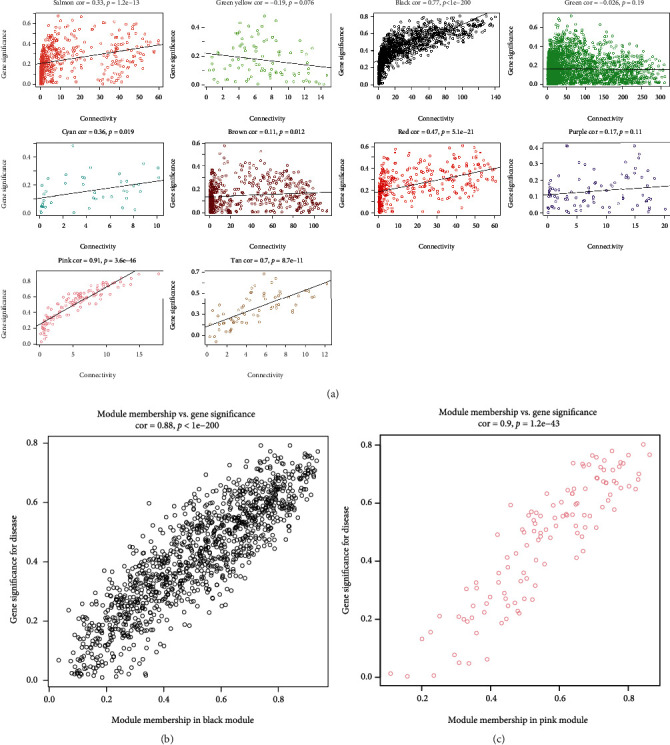
Key modules associated with CS. (a) Scatter plot of relationship between gene significance and gene connectivity in each module. Scatter plot of module eigengenes related to CS in the black (b) and pink (c) modules. CS: cardioembolic stroke.

Figure 3(a) illustrated the correlation between the connectivity and gene significance (GS) within each module. The correlation between module membership (MM) and GS in the black module and the pink module found that both of the *p* values were lower than 0.01, which demonstrates that these two modules are significantly related to CS. The correlation between MM and GS in the black module and the pink module was depicted in Figures 3(b) and 3(c). Considering the screening criteria of ∣MM | >0.9 and ∣GS | >0.4, it was determined that there are 18 core genes in the black module and one core gene in the pink module.

### 3.5. Screening of Differentially Expressed Genes (DEGs)

**Figure 4:**
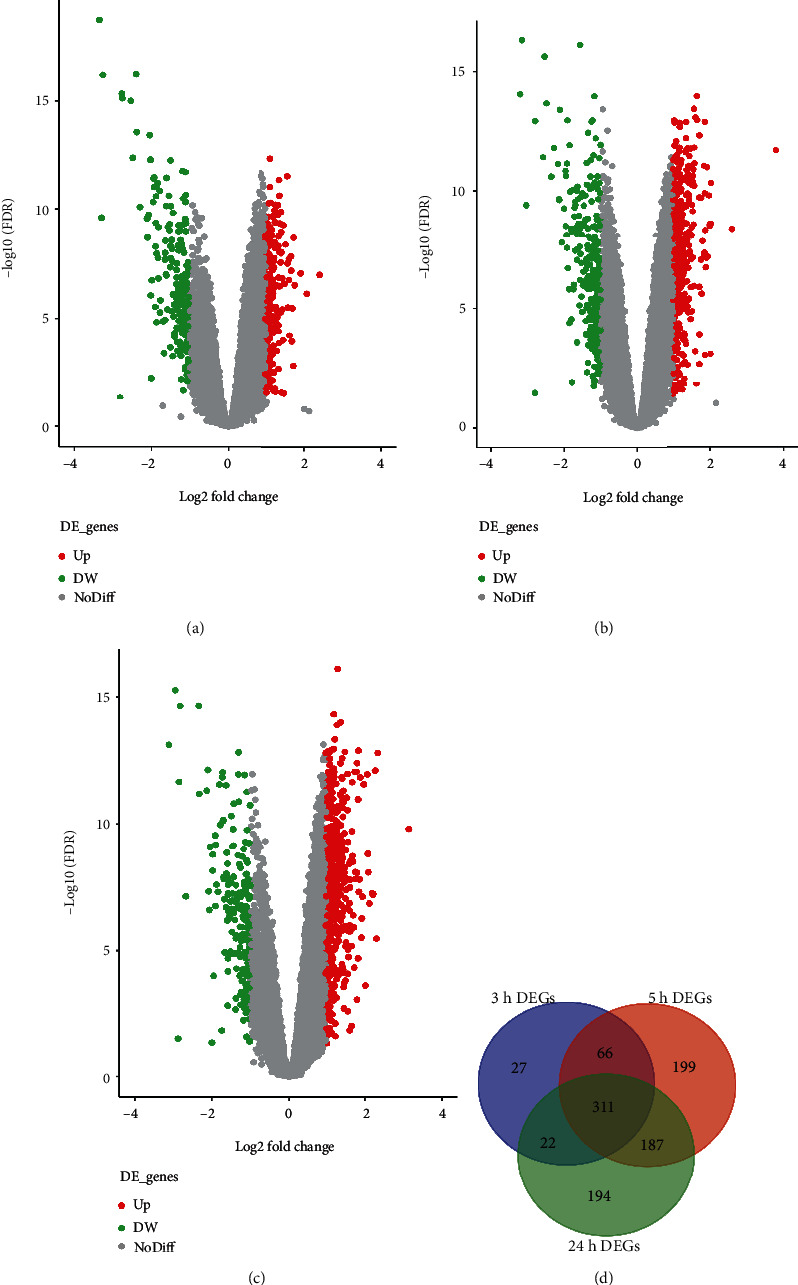
Screening of CS-related DEGs. (a) Volcano plot representing DEGs at 3 h following CS. (b) Volcano plot representing DEGs at 5 h following CS. (c) Volcano plot representing DEGs at 24 h following CS. (d) Venn diagram of DEGs at different time following CS. CS: cardioembolic stroke; DEGs: differentially expressed genes.

**Figure 5:**
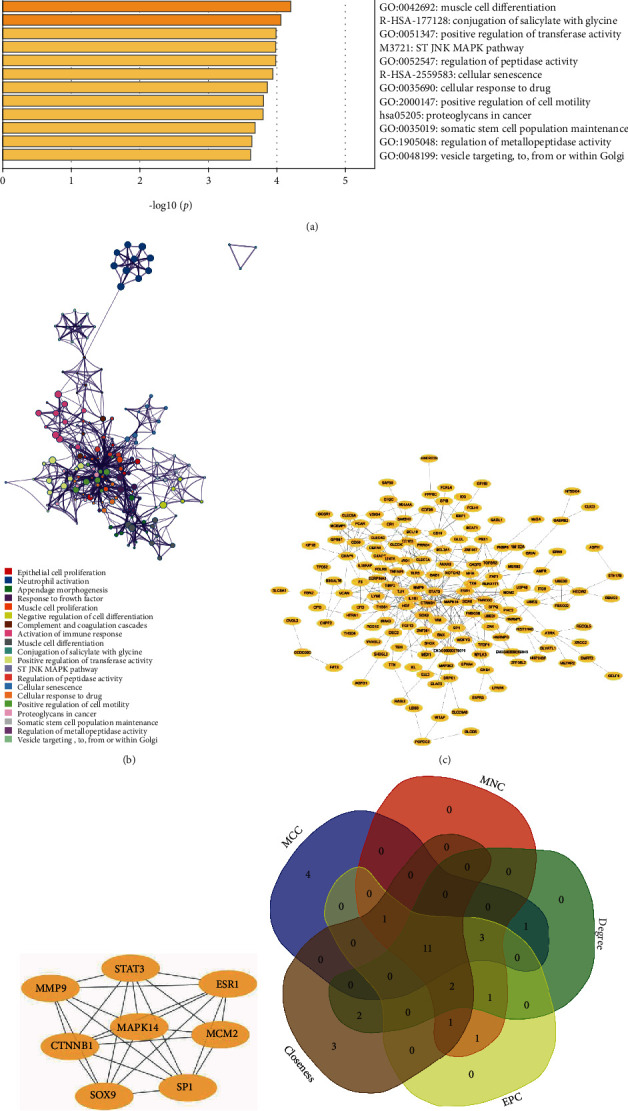
The identification of key genes in the CS-related DEGs. (a, b) The functional enrichment analysis of DEGs using Metascape. (c) Visualization of the PPI network of common DEGs by Cytoscape. (d) MCODE identified the most significant module of common DEGs. (e) Intersecting genes selected as key genes by using five algorithms in cytoHubba. CS: cardioembolic stroke; DEGs: differentially expressed genes; PPI: protein-protein interaction; MCODE: Molecular Complex Detection.

Figures 4(a)–4(c) illustrate the volcano plots of DEGs in healthy individuals and CS patients at 3 h, 5 h, and 24 h postonset obtained from the GSE58294 dataset by using GEO2R. After performing intersection, it was found that 311 genes in total were coexpressed at these three time points (Figure 4(d)). After conducting enrichment analysis on these coexpressed differential genes in the Metascape database, we found that these genes were enriched in neutrophil activation, activation of immune response, regulation of peptidase activity, and JNK/MAPK pathway, and so forth (Figures 5(a) and 5(b)).

### 3.6. Screening of Key Genes in DEGs

The abovementioned 311 coexpressed differential genes were subjected to PPI analysis in the STRING database and were visualized by using Cytoscape 3.7.2 (Figure 5(c)). The most significant subnetwork module screened out by using MCODE plug-in was displayed in Figure 5(d).

Subsequently, we used cytoHubba to screen out the hub genes from the DEGs, which were closely relevant to CS. The hub genes derived from the 5 algorithms in cytoHubba were listed in Table 1. The top 20 genes obtained from the 5 algorithms were intersected and 11 key genes were obtained (Figure 5(e)).

### 3.7. Hub Genes Associated with CS

**Figure 6:**
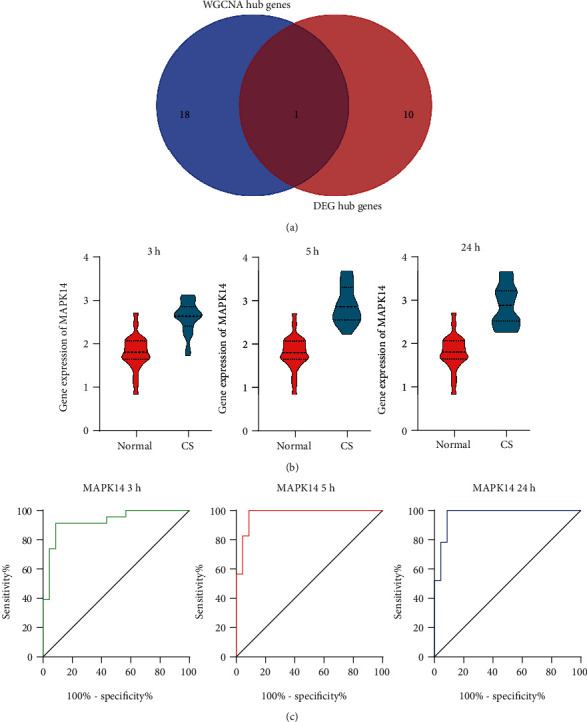
The identification of MAPK14 as a biomarker for CS. (a). Final overlap of WGCNA hub genes and DEG hub genes. (b). Violin plots representing expression of MAPK14 in 3 h, 5 h, and 24 h following CS were all significantly increased compared to normal individuals in dataset GSE58294. (c). Receiver operating characteristic (ROC) curves for blood MAPK14 in 3 h, 5 h, and 24 h following CS in dataset GSE58294. MAPK14: mitogen-activated protein kinase 14; CS: cardioembolic stroke; WGCNA: weighted gene coexpression network analysis; DEGs: differentially expressed genes.

The intersection of the core genes screened from WGCNA, and the key genes selected from DEGs yielded the mitogen-activated protein kinase 14 (MAPK14) gene, as seen in Figure 6(a). At 3 h, 5 h, and 24 h postonset, MAPK14 was notably upregulated in the CS patients group, as opposed to those in the normal control group. This result was indicative of the potential vital role played by MAPK14 in the progression of CS (Figure 6(b)). The ROC curve of CS diagnosed by the MAPK 14 expression level showed that the areas under the curve (AUC) at 3 h, 5 h, and 24 h postonset were 0.923, 0.934, and 0.941 (Figure 6(c)), respectively. These data suggested that MAPK14 is a promising biomarker for CS.

## 4. Discussion

In the current study, WGCNA was applied to analyze the gene expression data of the whole genome of CS blood samples, and 11 independent gene modules were obtained. Among these modules, the black module and the pink module were closely related to CS. GEO2R analysis revealed that the MAPK14 expression level was augmented at all three time points in CS, compared with the normal controlled group, and that MAPK14 was located in the black module. The ROC curve of CS based on the expression level of MAPK14 suggests that MAPK14 may be a potential newer biomarker that can be applied in the diagnosis of CS.

As a notorious disease, stroke is one of the leading causes of death and disability in adults [22]. However, the current preventive measures for stroke remain flawed: the prevention and treatment stress on stroke events that are mainly caused by cerebrovascular diseases, yet disregard ischemic stroke caused by cardiac diseases, such as atrial fibrillation and heart failure. Previous studies have found that the proportion of cardioembolic stroke (CS) events accounts for around 20% in all clinical ischemic stroke events and that its incidence increased significantly with aging, leading to notably higher mortality, disability, and recurrence rates than other types of stroke [6]. The continuous effort to find out the risk factors of CS is especially important, so are the active prevent and accurate diagnosis.

Accumulating evidence in recent years found that it is essential to construct a gene coexpression network within the framework of the current exploratory research, because such effort can help identify the important modules and genes related to disease [23]. The mining of marginal fold change genes remains challenging for traditional analysis at the gene level [24]. Nonetheless, the idea of mining genes by using the WGCNA analysis system provides a benefiting complement to the gene-level analysis [25]. WGCNA has been successfully applied in exploring the mechanism underlying diseases, diagnosis, prognosis prediction, and so on. The strong correlations in the results of WGCNA would remain intact or be less affected after the processing of power function. Meanwhile, the weak correlations would be markedly reduced power processing". These characteristics jointly contribute to better restore the entire physiological process in which the genes are involved. By contrast, the conventional clustering method cannot achieve such effect. Therefore, the results obtained from WGCNA possess higher credibility.

After WGCNA coexpression network analysis of CS and the investigation of hub genes, we found that MAPK14 is the DEG closely associated with CS. However, the exact mechanism underlying MAPK14 affects the biological behavior of neural cells in the onset and development of CS has not been clarified. In the present study, we make efforts to interrogate the correlation between genes and biology from four aspects, including the categories of BP, CC, MF, and KEGG signaling pathway analysis, thereby verifying the significant functional pathways in CS. Our results suggested that the DEGs of CS is mainly involved in molecular functions, biological processes, and signaling pathways, such as inflammatory response, immune response, cytokine interaction, and JNK pathway, all of which might be related to the occurrence and development of CS.

MAPK14 is a member of the MAP kinase family. As an integration checkpoint of various biochemical signals, MAP kinases are implicated in various processes, such as cell proliferation, differentiation, transcriptional regulation, and development [26]. Such kinase can be activated by various environmental stresses and proinflammatory cytokines [27]. Evidence confirmed that p38/MAPK plays a key role in the formation of cerebral edema [28, 29]. MAPK14 is widely present in the brain tissue, hence raises the hypothesis that it can reflect the degree of change in brain tissue concentration by detecting its concentration in the blood. Since the pathway can be activated by a variety of stress environments, subsequently damaging the human body by mediating inflammatory reactions and apoptosis, there exists a vital link between brain injury and the activation of p38MAPK pathway.

In the event of acute cerebral infarction, the acute ischemic injury in the brain tissue triggers detrimental cascades such as excitatory amino acid toxicity and inflammatory response, thereby reinforcing the activation of p38MAPK pathway in ischemic brain injury [30]. It plays a key part in the biological process of stress-induced neuronal death and has been confirmed that inhibition of p38 MAPK in vivo and in vitro significantly alleviated ischemic injury to brain tissue. To validate the bioinformatics analysis results acquired in this study, we plotted the ROC curve to measure the capacity of MAPK14 in the diagnosis of CS. The results showed that MAPK14 gene was significantly associated with the outcome of CS patients.

However, this study is not without certain limitations. Firstly, only the ROC curve was used to predict the diagnostic value of MAPK14 gene. Secondly, the sample size in the current study was relatively small. Thirdly, validation tests were not conducted and the severity data of CS were not included. All these shortcomings warrant further experimental studies in the future. The clinical efficacy of these biomarkers must be further explored in patients with CS. Further research is needed to examine the underlying mechanism and the related pathways of these genes.

In a nutshell, this study used WGCNA to screen the modular genes in the blood samples of CS patients, and the results showed that the expression level of MAPK14 in CS patients was notably higher than that in normal people. Such finding of MAPK14 is expected to shed light on a new diagnostic marker and therapeutic target for CS and provide theoretical basis for the molecular mechanism research that follows.

## 5. Conclusion

In this study, MAPK14 was screened out as the differential expressed gene in CS by using WGCNA. MAPK14 may participate in the pathogenesis of CS through immune regulation, inflammatory response, and JNK signaling pathways. This study provides promising opportunity to use MAPK14 as the biological marker for the diagnostic of CS.

## Figures and Tables

**Table 1:** The top 20 genes in the PPI network of coexpressed differentially expressed genes were identified by using the five algorithms in cytoHubba. MCC: maximal clique centrality; MNC: maximum neighborhood component; EPC: edge percolated component.

Gene name	MCC	Gene name	MNC	Gene name	Degree	Gene name	EPC	Gene name	Closeness
STAT3	1087	MMP9	22	STAT3	26	MMP9	62.732	STAT3	78.1
MMP9	1011	STAT3	21	MMP9	23	STAT3	62.774	MMP9	75.65
CTNNB1	992	CTNNB1	16	CTNNB1	20	CTNNB1	61.907	CTNNB1	73.06667
ESR1	922	MAPK14	13	MAPK14	19	MAPK14	61.571	MAPK14	72.31667
MAPK14	908	SOX9	13	ESR1	16	SOX9	61.536	ESR1	70.43333
SP1	877	ESR1	12	SOX9	15	ESR1	60.606	SOX9	68.06667
SOX9	782	HGF	12	HGF	15	HGF	60.445	SP1	67.35
CD59	735	TLR5	12	SP1	14	TLR5	58.117	MDM2	67.11667
CKAP4	733	SP1	11	TLR5	13	SP1	59.583	HGF	66.31667
CLEC4D	728	THBS1	11	CD59	13	THBS1	58.92	TLR5	64.01667
FCAR	722	CD59	10	MDM2	13	CD59	53.507	THBS1	63.13333
CLEC5A	722	CD163	10	THBS1	12	CD163	55.975	CD163	61.26667
GPR97	720	CKAP4	9	CD19	12	CKAP4	52.89	TJP1	60.9
MCEMP1	720	TIMP2	9	CLEC4D	11	TIMP2	55.794	VIM	60.86667
HGF	159	MDM2	8	CD163	10	MDM2	57.54	CD19	60.75
MDM2	153	CLEC7A	8	CKAP4	10	CLEC7A	53.965	FGF13	60.48333
THBS1	127	FGF13	8	VCAN	10	FGF13	57.717	TXN	60.18333
TLR5	103	ARG1	8	TJP1	10	ARG1	55.686	CLEC7A	59.38333
FGF13	103	SERPINA1	8	TIMP2	9	SERPINA1	54.683	ARG1	59.18333
TIMP2	72	VCAN	7	CLEC7A	9	VCAN	53.968	NOTCH2	58.98333

## Data Availability

All of the data used in current study are available upon reasonable request.
